# Successful preimplantation genetic testing for fibrodysplasia ossificans progressiva: a case report

**DOI:** 10.1186/s13256-024-04504-4

**Published:** 2024-04-26

**Authors:** Sughashini Murugesu, Benjamin P. Jones, Paul Serhal, Jara Ben-Nagi

**Affiliations:** 1 Queen Charlotte’s and Chelsea Hospital, Imperial College NHS Trust, Du Cane Road, London, W12 0HS UK; 2 Department of Surgery and Cancer, Imperial College London, London, W12 0NN UK; 3 Centre for Reproductive and Genetic Health, 230-232 Great Portland Street, London, W1W 5QS UK

**Keywords:** Fibrodysplasia ossificans progressive, Preimplantation genetic testing for monogenic disorder, Karyomapping

## Abstract

**Purpose of the study:**

Fibrodysplasia ossificans progressiva (FOP) is a rare autosomal dominant condition that leads to significant disability and morbidity, characterised by the formation of heterotopic hard tissues within connective tissues. The condition has an incidence of approximately one per two million people worldwide. There is no known single effective treatment available for FOP. We report the world’s first case of a healthy infant born following in vitro fertilisation (IVF) and preimplantation genetic testing for monogenic disorder (PGT-M) using Karyomapping for FOP.

**Case presentation:**

A 30-year-old Caucasian female with FOP presented with her partner seeking IVF with PGT-M to achieve a healthy pregnancy with an embryo unaffected by FOP.

**Methods:**

The couple underwent IVF and PGT-M using Karyomapping as the testing method. A multi-disciplinary team approach was utilised in planning this case, considering the additional risks of oocyte retrieval, pregnancy and childbirth in women with FOP.

**Main findings:**

The oocyte retrieval was covered with a 5-day course of prednisolone to reduce the risk of a localised inflammatory reaction, which could result in subsequent heterotopic ossification. This was subsequently weaned down with reducing doses every two days. The patient underwent uncomplicated oocyte retrieval, yielding 12 mature oocytes. Following intracytoplasmic sperm injection (ICSI), ten zygotes having two pro-nuclei were cultured, and six underwent trophoectoderm biopsy and vitrification 5–6 days after retrieval. PGT-M via Karyomapping revealed four out of six (66.7%) of blastocysts were not carriers of the maternal high-risk FOP allele. In total, the patient had three separate embryo transfers. Pregnancy was achieved following the third frozen embryo transfer, which went to 37 weeks’ gestation, and delivered by Caesarean section. The baby was born in excellent condition and is unaffected by FOP.

**Conclusion:**

IVF/ICSI and PGT-M using Karyomapping was successfully implemented to identify embryos carrying the high-risk FOP allele resulting in a healthy livebirth.

## Introduction

Fibrodysplasia ossificans progressiva (FOP) is a rare autosomal dominant condition that leads to significant disability and morbidity, characterised by the formation of heterotopic hard tissues within connective tissues [1, 2]. The disorder is extremely rare, with an incidence of approximately one per two million people worldwide [3]. There is no racial, ethnic, gender or geographic predilection for FOP incidence [4]. At the present time, there is no known single effective treatment available for FOP [5].

FOP is caused by a heterozygous gain of function mutation in the ACVR1/ALK2 gene (Activin receptor IA/activin-like kinase 2 (ACVR1; also known as ALK2)) located on chromosome 2, both in the sporadic and inherited cases. The outcome affects the bone morphogenetic proteins (BMP) signalling pathway. This ACVR1/ALK2 gene encodes for a transmembrane serine/threonine kinase receptor ALK2, which binds with the BMPs present in the bone matrix [6] leading to the development of heterotopic bone in skeletal muscle. Most cases of typical FOP show a nucleotide substitution in codon 617 (guanine->adenine) of the ACVR1/ALK2 gene [7] which changes the amino acid arginine to histidine in the corresponding protein [8].

Patients with FOP are usually misdiagnosed early in life before the appearance of heterotopic ossification and thus undergo diagnostic procedures that can exacerbate formation of heterotopic bone and accelerate the extensive and lifelong disability [9, 10]. Disease progression is episodic, however, the nature of pathology results in a cumulative disability. There is progressive immobilization of the chest wall, limbs and jaw by early adulthood, and by the third decade of life, most patients are wheelchair bound [11, 12]. Median life expectancy is approximately 40 years, and patients usually die from complications of thoracic insufficiency syndrome. [13, 14]

Reproductive aspirations and planning can often present a dilemma for individuals with FOP. It is possible for women with FOP to be pregnant, owing to the absence of smooth muscle involvement in this condition, and a number of cases of childbirth have been reported in the medical literature [15]. However, unique and difficult management challenges require consideration in such cases to the patient, family members and health-care providers [10]. Given the debilitating nature of the condition and the autosomal dominant inheritance, a major concern for a couple, where one partner has FOP, is the 50% chance of the child inheriting the condition. Following the identification of the mutation in the ACVR1/ALK2 gene, it is now possible to mitigate the risk of inheritance by using in vitro fertilization (IVF) with preimplantation genetic testing for monogenic disorder (PGT-M). For this condition, Karyomapping and “phasing analysis” can be used to identify the inheritance of the high-risk FOP allele [16, 17]. Following embryo biopsy, deoxyribonucleic acid (DNA) extracted from cells is subsequently subjected to whole genome amplification and analysed using a Karyomapping array capable of interrogating a large number of single nucleotide polymorphisms (SNPs) [18] needed for phasing. The aim of this case report is to describe the feasibility and effectiveness of using PGT-M and IVF in FOP.

## Case report

A 30 year-old Caucasian woman with FOP and her partner presented to the Centre for Reproductive and Genetic Health, London, UK to undergo PGT-M treatment. The woman carried a heterozygous pathogenic variant c.774G>T (p.Arg258Ser) in the ACVR1 gene and had a relatively mild phenotype, having only had two flare ups of FOP prior to presentation at the clinic. A multi-disciplinary team (MDT) approach was utilised to optimally organise this patient’s care. The woman was counselled comprehensively regarding the risk of the condition flaring up during ovarian stimulation, oocyte retrieval and pregnancy.

## Methods

Controlled ovarian stimulation was undertaken with a gonadotropin releasing hormone (GnRH) antagonist protocol after pre-treatment with 12 days norethisterone 5mg twice a day. She was triggered on day 12 with peak oestradiol level 6890 mIU/ml. A GnRH agonist trigger was given 37hr prior to transvaginal ultrasound-guided retrieval. Intramuscular injections were specifically not given in view of risk of inflammatory reaction and subsequent ossification.

To reduce the risk of a localised inflammatory reaction which could result in subsequent heterotopic ossification, the oocyte retrieval was covered with a five-day course of prednisolone [19]. This was subsequently weaned down with reducing doses every two days. The patient underwent uncomplicated oocyte retrieval, yielding 12 mature oocytes. The mature oocytes were retrieved and subsequently underwent intracytoplasmic sperm injection (ICSI). Ten oocytes developed two pronuclei and were cultured to blastocyst stage. Six blastocysts of the following morphology 6B+B+, 2 of 6 B+B−, 6B−B+, 6B−B−,6B−C had trophectoderm biopsy on day five after retrieval and underwent genetic analysis. The embryo morphology and grading has been previously described. [18]

In the workup towards PGT-M, DNA was extracted from the intended parents, using the QIAamp DNA Mini kit (Qiagen, USA). Following this, the DNA samples were processed by the Infinium Karyomapping Assay (Illumina, USA) where the genomic region spanning the ACVR1/ALK2 gene was assessed for SNP informativity and call rates using an Illumina dedicated software (BlueFuse Multi). Given that no samples were available from additional affected family members, Karyomapping was combined with direct interrogation of the mutation site. This involved PCR amplification of a fragment of DNA encompassing the mutation followed by minisequencing using the SNaPshot™ multiplex kit (Thermofisher, USA) to reveal whether the mutant or normal allele was present. Once direct interrogation of the mutation was applied to the embryos, a suitable diagnosed embryo is chosen as the reference and phasing is done for the rest of the embryos produced by the patients. The final diagnosis was then based upon a combination of Karyomapping and direct mutation detection.

Samples derived from embryos (that is biopsy specimens) were processed in a clean environment at Genesis, Genetics, UK. The first step in this process was cell lysis, followed by whole genome amplification using SureMDA (Illumina, USA), according to the manufacturer’s instructions. The biopsied specimens were incubated at 30°C for 2 hours, after which enzyme inactivation was carried out at 65°C for 5 minutes. Gel electrophoresis using a 1% agarose gel was then used to assess the amplification. An aliquot of MDA product was then used for the Infinium Karyomapping Assay, following the standard protocol. The assay involved several steps where the amplified DNA from the embryo specimens together with parental and reference genomic DNA were prepared and hybridized to the HumanKaryomap-12 BeadChip SNP array. This included a second round of whole genome amplification, fragmentation, precipitation, resuspension and hybridization of DNA to the BeadChip, washing of the BeadChip and ‘extend and stain’ of the BeadChip. The final stage of the Infinium Karyomapping assay involved scanning of the BeadChip SNP array slides with the Illumina iScan System. The SNP data were then imported into BlueFuse Multi (Illumina) to be analysed. The entire process from sample receipt until reporting of results had a duration of approximately 24 hours and yielded data on the inheritance of the FOP allele as well as information concerning aneuploidy. The outcome of Karyomapping found one blastocyst was a carrier of the autosomal dominant inherited allele, and thus would be affected by FOP. Four embryos did not have the high-risk allele and thus would produce children unaffected by FOP.

A successful pregnancy was achieved following the third medicated embryo transfer. The patient was under a specialist obstetric medicine team and the pregnancy advanced to 37 weeks’ gestation, delivered via Caesarean section under general anaesthetic due to maternal FOP. A male child was born, weighing 2740g, phenotypically normal with no features of FOP. The child has developed normally meeting all paediatric assessments, reaching all milestones, to the current age of 12 months (at the time of preparation of this article).

## Discussion

This case report is the first to demonstrate that Karyomapping can be used for PGT-M of this autosomal dominant condition. The genotypes of both parents are required to identify the distinct set of SNP markers associated with the relevant gene [20]. The SNP markers present in the parental chromosomes combined with direct interrogation of the mutation site, were used to determine whether the embryos biopsied were carriers of the mutation by linkage analysis. Natesan *et al.* 2014 reported on the accuracy of genome-wide Karyomapping in identifying single gene defects in preimplantation human embryos [21].

One of the main advantages of Karyomapping is the greatly reduced workup time and laboratory effort required to prepare for a PGD cycle [22]. This is especially advantageous where ovarian reserve may be lower, PGD outcome is highly dependent on oocyte yield following ovarian stimulation [23–25]. Ben-Nagi *et al.* 2017 reported a 63% ongoing pregnancy rate per embryo transfer in a study of a total of 81 embryo transfers [18]. Karyomapping potentially offers higher pregnancy rates than other PGT-M methods due to the additional detection of aneuploidy and selection at blastocyst stage.

**Fig. 1:**
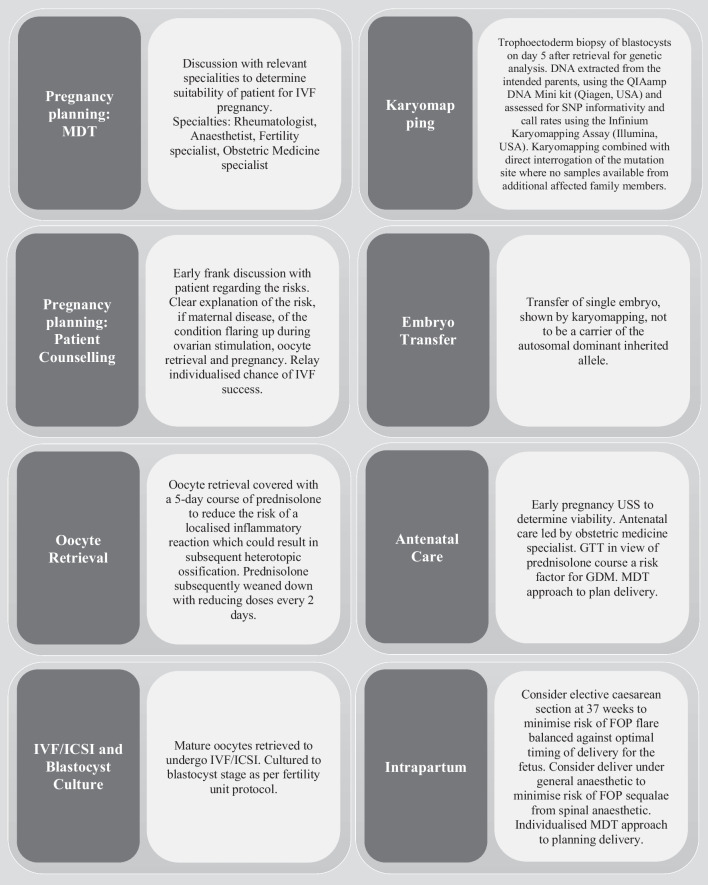
Guidance for specialists managing FOP patients during the preconception, antenatal and intrapartum periods. (Figure created by author S.M). *MDT* Multi-disciplinary Team, *IVF* In Vitro Fertilisation, *ICSI* Intracytoplasmic Sperm Injection, *SNP* Single Nucleotide Polypeptide, *USS* Ultrasound Scan, *GTT* Glucose Tolerance Test, *GDM* Gestational Diabetes, *FOP* Fibrodysplasia Ossificans Progressiva

FOP is a rare condition, where there are significant implications resulting from pregnancy and childbirth. If pregnancy is contemplated in a woman with FOP, pre-pregnancy counselling is essential because FOP is an autosomal dominant disorder with complete penetrance [4, 10]. Figure 1 outlines a flow chart of considerations in caring for women with FOP through a planned pregnancy. The case we describe, where there has been a successful outcome in counselling a couple that their child will be unaffected by FOP, has changed the narrative for these individuals. This treatment offers the opportunity to ensure the FOP allele is not inherited and thus alleviates some of the anxiety that may surround such a couple aiming to start a family.

It is important to note that aside from the child having a 50% chance of inheriting the FOP allele, there are additional complications that may arise in women with FOP during pregnancy, which may have an impact upon the fetus. In our case, the pregnancy advanced to term, but in pregnancies amongst women with FOP there is increased risk of prematurity, fetal distress and the risk of complications during general anaesthesia [10]. If a flare were to occur during pregnancy, the chronic use of high-dose glucocorticoids may increase the risk of gestational diabetes [26], whereas the use of non-steroidal anti-inflammatory drugs may promote premature closure of the ductus arteriosus, leading to fetal pulmonary hypertension, if used in close proximity to delivery [27]. Thus, despite PGT-M mitigating the risk of vertical transmission of FOP, it is important to for women to undergo comprehensive reproductive counselling including the risks associated with maternal FOP on pregnancy. Careful consideration with MDT input including obstetric medicine, rheumatology, paediatric, and anaesthetic input is essential.

## Conclusion

We report the first successful application of PGT-M by means of trophectoderm biopsy of blastocyst stage embryos and Karyomapping analysis, to avoid vertical transmission of the autosomal dominant high-risk allele resulting in FOP. We outline the importance of an MDT approach in optimising the care of maternal FOP.

## Data Availability

Not applicable.
